# Macaques preferentially attend to visual patterns with higher fractal dimension contours

**DOI:** 10.1038/s41598-019-46799-0

**Published:** 2019-07-22

**Authors:** Kelly R. Finn, James P. Crutchfield, Eliza Bliss-Moreau

**Affiliations:** 1 Animal Behavior Graduate Group, University of California Davis, Davis, United States; 2 Department of Population Health and Reproduction, School of Veterinary Medicine, University of California Davis, Davis, United States; 3 California National Primate Research Center, University of California Davis, Davis, United States; 4 Complexity Sciences Center, University of California Davis, Davis, United States; 5 Department of Physics, University of California Davis, Davis, United States; 6 Department of Psychology, University of California Davis, Davis, United States

**Keywords:** Psychology, Complexity, Visual system

## Abstract

Animals’ sensory systems evolved to efficiently process information from their environmental niches. Niches often include irregular shapes and rough textures (e.g., jagged terrain, canopy outlines) that must be navigated to find food, escape predators, and master other fitness-related challenges. For most primates, vision is the dominant sensory modality and thus, primates have evolved systems for processing complicated visual stimuli. One way to quantify information present in visual stimuli in natural scenes is evaluating their fractal dimension. We hypothesized that sensitivity to complicated geometric forms, indexed by fractal dimension, is an evolutionarily conserved capacity, and tested this capacity in rhesus macaques (*Macaca mulatta)*. Monkeys viewed paired black and white images of simulated self-similar contours that systematically varied in fractal dimension while their attention to the stimuli was measured using noninvasive infrared eye tracking. They fixated more frequently on, dwelled for longer durations on, and had attentional biases towards images that contain boundary contours with higher fractal dimensions. This indicates that, like humans, they discriminate between visual stimuli on the basis of fractal dimension and may prefer viewing informationally rich visual stimuli. Our findings suggest that sensitivity to fractal dimension may be a wider ability of the vertebrate vision system.

## Introduction

Animals mine their environments for actionable information via their sensory systems in order to navigate their habitats. However, the diversity of raw physical phenomena in nature presents an enormous variety of potential sensory stimuli. The question is, paraphrasing Bateson^1^, which signals are actually relevant and make a difference? What differences in the spatial or temporal configurations of physical objects and events in an environment matter to an individual animal? Through evolution and development, sensory systems become tuned to process environmental information that is important to individuals^2,3^. As a result, what information an animal extracts from the environment via its sensory systems is related to the statistical regularities of the natural environment in which it lives^4–7^. Pattern recognition allows animals to readily detect stimuli relevant to allostasis (e.g. food, shelter, predators, or mates) and ignore stimuli that are not important.

For most primates, vision is the dominant sensory modality^8,9^, and the primate eye is thought to be proficient at sampling informative signals existing in or caused by natural phenomena^10^. Acute vision is critical for most primates, allowing them to physically navigate their environments, which often include irregular shapes and rough textures (e.g., jagged terrain and canopy outlines). Therefore, we hypothesized that nonhuman primates should be able to rapidly distinguish between visual stimuli that vary in terms of how much and what type of information is in them. We investigated the evolution of visual information processing by evaluating rhesus macaques (*Macaca mulatta*), a monkey species which shares a fairly close evolutionary history with humans^11^. Specifically, we evaluated the ability of the monkeys to differentiate between visual stimuli in which the extant information varies in terms of fractal dimension. While there is no single quantitative measure that alone fully describes the way in which information is distributed and organized in a pattern, fractal dimension is a well-defined descriptor of statistical scaling properties (among other properties such as global unpredictability and structural complexity^12^) to which primate visual systems may be sensitive. Indeed, fractal dimension has a well-established association to self-similar shapes found in natural environments^13–15^.

Straight edges and smooth surfaces are the standard objects in traditional geometry, but they are the exception in nature. Nature is filled with rough, irregular patterns, from the texture of a rock to a jagged mountain landscape. Such nonuniform shapes were once considered mathematically indescribable. Eventually, however, geometry was extended to describe the irregularity of natural patterns^13^, creating the ability to more precisely study how individuals process visual patterns that more closely mirror those arising in natural environments. One such method quantifies the less regular visual patterns in natural scenes by evaluating their fractal dimension^13,16^. Fractal dimension (*d*_*f*_) measures self-similarity – the amount of detail in an object that is consistent as one varies magnification across a range of scales. Fractal dimension corresponds to human self-reported judgements of how ‘complex’^17^ or ‘rough’^16^ a visual stimulus is – the higher the *d*_*f*_, the rougher or more complicated people report a stimulus to be.

Fractal geometry so well represents a property of natural patterns to which humans are attuned, that it revolutionized computer-generated graphics—fractal generation algorithms have been used for over three decades to create realistic appearing images of trees, clouds, mountains, islands, and even entire planets (e.g. in animated movies, video games, or other computer-generated imagery). A classic example of a fractal description of the natural world is the measurement of a coastline^18^—as the resolution of measurement becomes finer, it captures smaller cliffs and coves, yielding longer and longer measurements of coastline length. In the extreme case of arbitrarily fine measurements, for a compact object like the island of Great Britain the measured coastline length increases without bound. Ultimately, one realizes that measured lengths are entirely dependent on the scale at which they are measured. However, fractal dimension, specifically how the total measurement scales with the measurement resolution, quantifies unique information about the object being measured. Expressly, *d*_*f*_ quantifies this characteristic of irregularly shaped objects as a measure of roughness to describe the shape^18^. Fractal dimension is similar to the common notion of ‘dimension’ of an object in mathematical space (e.g. lines are one-dimensional and planes are two-dimensional), except the *d*_*f*_ can be fractional, filling space at some fraction that is not an integer value^18^. Thus, irregular curves will have a *d*_*f*_ between 1 and 2 and the west coastline of Great Britain has a *d*_*f*_ of 1.25^18^. Since fractals are used to model the natural world in ways humans deem exceptionally realistic, it stands to reason that *d*_*f*_ captures unique variation in the physical shape of objects to which the sensory systems of humans, and possibly of other species, are sensitive.

The human vision system is well-tuned to recognize and discriminate between visual stimuli of varying *d*_*f*_^17,19,20^. Visual psychophysics experiments conducted with people have established sensitivity to *d*_*f*_ for curves (i.e., contiguous sets of points) and boundary contours (i.e., the edge shape of an object). For instance, when asked to rate one-dimensional curves and two-dimensional surfaces from smoothest to roughest, human estimates were highly correlated to the image’s *d*_*f*_^16^, demonstrating that fractal dimension is a good index for a human’s perceptual notion of ‘roughness’. Similarly, *d*_*f*_, along with the number of self-similar scales (i.e., the number of reiterations of the same shape at different scales within a given image), were good predictors of how humans rated generated fractal curves with regard to ‘how complex it appeared’^17^. People are even better able to distinguish differences in roughness indexed by *d*_*f*_ than standard statistical estimations for *d*_*f*_ applied to the same curve data^21^. Kumar and colleagues^21^ presented subjects with jagged lines that differed in *d*_*f*_ and asked them to judge how rough the curve appeared relative to a reference image. Participants were better able to discriminate differences in *d*_*f*_ with their judgements of ‘roughness’ than 4 out of 5 algorithms which estimated the Hurst exponent of the curves (a proxy for *d*_*f*_). These studies demonstrate the remarkable capacity of the human vision system to identify a type of visual complexity that is indexed by fractal dimension. Whether or not primates other than humans are sensitive to differences in fractal dimension in visual stimuli, however, has not been investigated. Sensitivity to *d*_*f*_ in species other than humans would have clear implications for the evolution of visual systems within an ecological context, since the natural physical environment, replete with patterns well described by *d*_*f*_, is a strong driver to the evolution and development of sensory systems^2,3^.

Beyond being able to detect variation in visual stimuli as measured by *d*_*f*_, people also self-reported variation in perceptual experiences while viewing images that varied by *d*_*f*_. For instance, when people viewed pictures with fractal boundary contours, i.e. shape edges (both filled and empty) that varied from *d*_*f*_ = 1.2 to *d*_*f*_ = 1.8, they were more likely to identify “nameable shapes” and more frequent “popping out” of objects in lower dimension images^20^. (The latter refers to profiles, animals, and the like, that appear in a spontaneous way similar to the pastime of looking for objects in cloud formations or to identifying objects in ink-blot Rorschach tests). People also report aesthetic preferences to curves and contours with certain *d*_*f*_, though preferences for specific *d*_*f*_ vary across studies that differ in stimuli types^22–25^. In forced-choice experiments where people were asked to choose which of two images were most ‘visually appealing’, they showed a preference for mid-range *d*_*f*_ between 1.3–1.5^22–24^ when stimuli consisted of natural scenes, abstract art, and simulated fractal contours. However, Bies and colleagues^25^ reported that most humans preferred images with the highest *d*_*f*_ when stimuli consisted of mathematically exact fractals, where the exact same shape can be seen at different magnifications. This is in contrast to the previously reported preference to mid-rage *d*_*f*_ where stimuli were statistically self-similar fractals, which are more often seen in nature, where only statistical properties of the shape are preserved at different magnifications. These studies hint that beyond people’s ability to discriminate between images on the basis of *d*_*f*_, images of different *d*_*f*_ may elicit different types of cognitive or affective experiences.

Whether human sensitivity to the *d*_*f*_ of visual stimuli is an older evolutionary capacity of the primate brain is unknown. The prevalence of fractal-like patterns in nature^13^ makes it likely that the vision systems of some nonhuman animals are also sensitive to *d*_*f*_. We explored this possibility in rhesus monkeys (*Macaca mulatta*), who have visual systems that are very similar to humans^26,27^. Humans and macaques not only share common ancestry diverging in evolutionary time only approximately 25 million years ago^11,28^, but today commonly occupy the same or similar environments^29^. Comparing humans’ and monkeys’ abilities to detect and process complex patterns that are prevalent in nature opens these psychophysical investigations to an evolutionary scope. This allows us to more broadly consider how, over evolutionary time, sensory systems have evolved to detect information in an animal’s environment, and how nature’s designs have shaped pattern detection abilities of vision systems.

While several previous studies used fractal stimuli with macaques^30,31^, never before has their ability to distinguish between *d*_*f*_ of such stimuli been evaluated. That is, previous studies used images of colorful shapes produced by fractal algorithms as stimuli for visual memory studies, since computer-generated fractals offer effective means to create complicated and diverse trial-unique stimuli sets^30^, but they have not specifically evaluated how those stimuli are processed. In the present study, we presented monkeys with paired black-white images of simulated self-similar contours that varied from low *d*_*f*_ (*d*_*f*_ = 1.1) to high *d*_*f*_ (*d*_*f*_ = 1.9) and then measured their visual attention to the stimuli using noninvasive infrared eye tracking. We chose to use stimuli that varied in fractal dimension of *contours* since previous studies showed this to be an important quality in shape perception in humans^20,32^. A suite of variables related to visual attention, recorded with an infrared eye tracker, were analyzed to determine if monkeys can discriminate between images that vary in *d*_*f*_, and if attentional differences exist that could indicate viewing preferences to images with higher or lower *d*_*f*_.

## Methods

**Figure 1:**
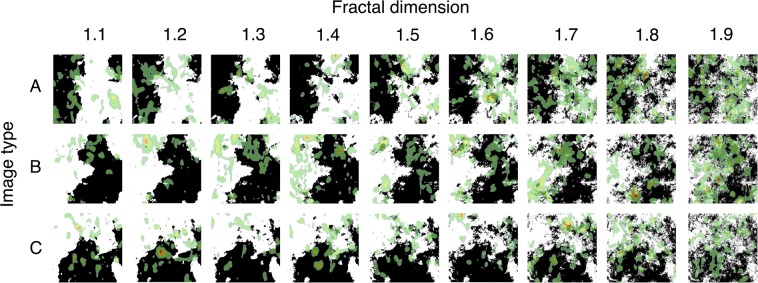
Distribution of visual attention on experimental stimuli. Visual attention data from all monkeys was aggregated for each stimulus and is depicted as a heatmap overlaid on each stimulus using the default heatmap/point-of-gaze map setting in the ASL Results Plus software (Applied Science Laboratories). Each heatmap was generated from the eye position data from one trial per monkey, when the stimulus was the left image and paired with itself. Warmer colors (red) indicate more visual attention that cooler colors (green). Stimuli, created in^23,33^, were generated from three different seed images (**A–C**), with contours of fractal dimension *d*_*f*_ from 1.1 to 1.9.

To determine if monkeys have the capacity to discriminate between visual stimuli that vary in *d*_*f*_, we presented adult male macaques with paired simulated self-similar black and white contours or ‘coastlines’^23,33^ and recorded their eye movement with an infrared eye tracker (see Fig. 1).

### Ethics statement

All experimental procedures were approved by the University of California, Davis, Institutional Animal Care and Use Committee in accordance with the National Institute of Health’s *The Guide*^34^.

### Experimental model and subject details

Seven adult male rhesus macaques (*Macaca mulatta*) (*Mean*_*ag*e_ = 11.14 years, *SD*_*age*_ = 0.59 years on their first days of testing), participated in the study. Monkeys were socially reared and lived in 0.5 acre field enclosures (each housing 60–120 monkeys) until adulthood when they were moved inside and paired with a compatible social partner. During the present experiment, monkeys were socially housed. Five of the seven were paired with another animal for 7-hours a day and one monkey was paired 24 hours per day with his pair-mate. One of the monkeys was paired 7-hours per day half of the testing period and awaiting a new pair-mate during the second half of the testing period. They were housed in standard adult macaque laboratory cages (111.4 cm width × 68.1 cm length × 92.1 cm height) in rooms that were maintained on a 12-hour light/dark cycle (lights on at 0600) at ~26 degrees C. Monkeys were fed Purina monkey chow twice daily, supplemented weekly with fresh fruit and vegetables, had access to water *ad lib*, and had daily enrichment (including toys, videos, forage, etc.).

### Fractal dimension perception task

#### Stimuli

Three sets of nine computer-generated images were used (binary images in^33^, see Fig. 1). Each image in a set is a version of the same ‘seed’ image and is generated with a contour fractal dimension (*d*_*f*_) of 1.1 to 1.9, increasing in d_f_ in increments of 0.1. Each image contained 50% white and 50% black to control for the density, contrast, and luminance of the images. We did not investigate *d*_*f*_ = 2.0 image stimuli since, though at “higher” fractal dimension, these images are uniform luminance fields and so have no discernible contours. They are, in other words, differently structured stimuli.

#### Procedure

Each monkey completed three task versions (81 trials per version) for a total of 243 trials per monkey. Each trial included two fractal images that were generated from the same seed image. Images of all fractal dimensions were matched with all other fractal dimensions, and pairs of images were presented in a randomized ordering. The procedure was optimized for animals who preferred to complete fewer trials per day by ending a version near half completion when trial completion speed dropped, and the second half of the version was completed on a second day. Each animal viewed stimuli of each fractal dimensions approximately the same number of times; but due to a coding error, not all stimuli were viewed exactly the same number of times. Each animal viewed 378 stimuli with *d*_*f*_ of 1.1, 1.3, 1.4, and 1.5, 376 for *d*_*f*_ of 1.2, 336 for *d*_*f*_ of 1.6, 392 for *d*_*f*_ of 1.7 and 1.9, and 390 for *d*_*f*_ of 1.8. Data was only recorded when monkeys looked at the computer screen – thus two monkeys had two fewer trials included in the final analysis because they did not look at the screen during two trials.

Animals completed testing while seated in a box chair with a slanted top (Crist Instrument Co., Inc., Damascus, MD). Their heads were restrained with thermoplastic face masks allowing for accurate eye tracking^35^. Testing occurred inside a sound-attenuating testing chamber (Acoustic Systems, Austin, TX; 2.1 m wide × 2.4 m tall × 1.1 m deep) with a white noise generator (60 dB) to mask outside noise. We used a noninvasive infrared eye-tracker (Applied Science Laboratories, Bedford, MA; model R-HS-S6), positioned 53.34 cm from the monkey’s eyes on a tripod to monitor eye movement. Detailed descriptions of animal training and calibration methods can be found in^35–37^.

**Figure 2:**
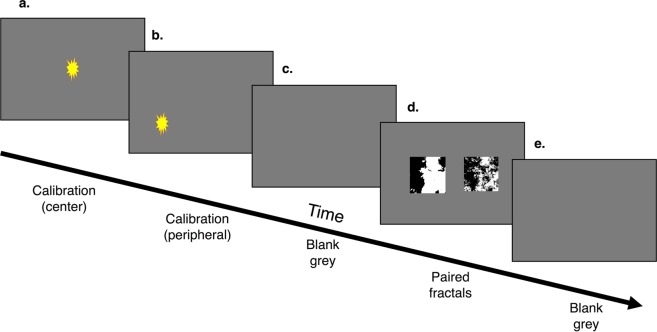
Overview of a trial. Each trial begins with (**a**) a self-paced center star for calibration which is rewarded with juice after a fixation, (**b**) a self-paced peripheral star for calibration which is rewarded with juice after a fixation, (**c**) five seconds of blank grey screen, (**d)** 10 seconds of the paired fractal stimuli, and (**e**) 5 seconds of blank grey screen.

Stimuli were presented on a wide-screen, color video monitor (60.96 cm diagonal; Gateway Inc., Irvine, CA; model LP2424) positioned at eye-level, 127 cm from the monkeys’ eyes. To ensure eye-tracker calibration and that the monkeys were engaged with the task, each trial started by requiring animals to look at and hold their attention on (i.e., fixate upon) a blinking star to receive a juice reward dispensed through a curved mouthpiece (Crist Instrument Co., Inc.; model # 5-RLD-00A) from an automatic juice dispenser (Crist Instrument Co., Inc.; model # 5-RLD-E3). It is important to note that rewards only occurred during these calibration steps, and the experimental steps which involved the stimuli were ‘passive viewing’ and not rewarded. In other words, monkeys could choose not to view the experimental stimuli and would receive the same rewards each trial during calibrations. The calibrations were self-paced, two fixation stars presented in sequence and each requiring a fixation of greater than 500 ms. After (a & b) two calibration slides (a & b, Figure 2) the monkeys were presented with (c) a 50% grey screen for 5 seconds, followed by (d) two paired fractal images on a 50% grey backdrop for 10 seconds. Each trial ended with (e) 5 seconds of a 50% grey screen. Stimuli were presented from a PC running Eprime 2.0 Professional software package (Psychology Software Tools, Pittsburgh, PA). See Fig. 2 for a schematic of one trial.

### Processing of eye-tracking data and statistical analysis

#### Infrared eye-tracking data

Visual attention data were recorded using the Eye-Trac 6.NET User Interface program (Applied Science Laboratories, Bedford, MA). Two measurements of visual attention were quantified using the standard settings in the ASL Results Plus software (Applied Science Laboratories): fixations and dwells. The parameters for each area of interest, *AOI*, was determined graphically using the ASL Results Plus software (Applied Science Laboratories) by drawing a rectangle around the placement of each image.

Within the Results Plus software, fixations were defined as small points where gaze within a 1° × 1° visual angle which remains static for at least 100 ms. Dwells were defined as visits to an area of interest, *AOI*, in this case a single fractal image, from entry to exit. Both fixations and dwells have an associated frequency and duration. We analyzed the total count, average duration, and total duration of fixations; the total count, average duration, and total duration of dwells. Having a repertoire of eye movements that include fixations and saccades (movements between fixations) and the ability to direct and hold gaze to stimuli of interest (dwells) is widespread across visually adept animals^38^. Further, nonhuman primates specifically share the same ventral and dorsal visual processing pathways as humans^39^, which are associated with information processing (or suppression of) during fixations and dwells. Consequently, we expect our macaque fixation and dwell data to be comparable to human data with regard to information processing and attention.

We also extracted the average dark-adapted pupil diameter on each stimulus for each trial, which is calculated from the average pupil diameter during each fixation on the *AOI*, weighted by the fixations’ durations, using the standard settings in the software. We normalized average pupil diameter by dividing the average pupil diameter for each *AOI* for each trial by the individual’s average pupil diameter across all images in all trials, to account for individual differences in pupil size and pupil reactivity^40^. Measuring pupil diameter is a robust cross-species tool because humans and macaques share similar neurological operation of the pupillary light reflex^41^. Indeed, much of what we know about the neural pathways and brain regions associated with cognition-related pupil responses in humans is actually based on monkey studies which investigated brain structures that have been phylogenetically conserved^42–49^. Additionally, pupil diameter has been used to index cognitive processing load and physiological arousal in macaques^40^. Humans and macaques are known to share autonomic nervous system responses to at least some affective processes; for example, autonomic arousal indexed by cardiac function vary by stimuli valence, mirroring the relationships found in humans^50^. Thus, we expect our pupil diameter data to be comparable to humans at least broadly in relation to both light reflex and general cognitive states such as attention, arousal, or high mental effort, which likely trigger similar autonomic responses across species.

Finally, we calculated each monkey’s attention bias for each trial, for one image compared to the other. That is, we computed a ratio of the total dwell duration on one image relative to the total dwell duration on both images of a given trial. This was calculated for each trial separately. This metric allowed for evaluation of preferential attention between stimuli of multiple *d*_*f*_, even when the two stimuli on a given trial are very close in *d*_*f*_. Therefore, fixation and dwell measures, pupil diameter, and attention bias provide distinct measures for different outcomes of visual attention.

#### Data analysis

We determined the best-fit distribution of the data using functions from the fitdistrplus package^51^ of R statistical software^52^. Visual inspection of the data, cumulative density function plots, and AIC and BIC scores converged, which led us to conclude negative binomial distributions provided the best fits for our data except for attention biases and pupil diameter, which were normally distributed. We then used the lme4 package^53^ to run generalized linear mixed effect models for the negative binomial and Gaussian families, respectively. We modeled attributes of fixations and dwells, and pupil diameter as outcome variables using subject as a random effect and including or omitting the *d*_*f*_ of the image as a predictor, and the *d*_*f*_ of the paired image as a second predictor and compared the AIC scores for the models. Best-fit models included all variables as predictors. The one exception was in the models with pupil diameter, where AIC of models with and without paired image *d*_*f*_ did equally well (difference of <1; we used the model without paired image *d*_*f*_ because it is a more logical fit). We modeled attention bias as an outcome variable using subject as a random effect, included and omitted the difference in *d*_*f*_ of the images as a predictor, and compared the AIC scores for the models; the best fit model included this predictor. We did not include trial number in statistical models, as all individuals completed three versions of the experiment, each of which presented paired stimuli in a randomized order to control for order effects.

## Results

### Fixations

**Figure 3:**
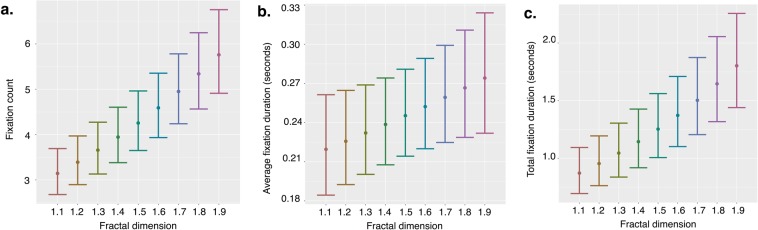
Fixation characteristics by fractal dimension**. (a**) Macaques fixated on images with greater *d*_*f*_ more times during a trial, (**b**) on average fixated longer during each fixation, and (**c)** spent a larger total duration of time fixating on images of greater *d*_*f*_. Estimated marginal means from the statistical model are depicted as points; error bars are the 95% confidence interval of the mean. Paired *d*_*f*_ is held at 0.5 (See Supplementary Fig. S1 for complementary figures using raw data).

As the *d*_*f*_ of the stimuli increased, so too did the number of fixations on each stimulus: *Estimate = *0.076, *Standard Error (SE) = *0.006, *z-score (z)* = 13.437, *p-value (p)* < 0.001 (see Fig. 3a). Monkeys also evidenced longer average fixations on images as the *d*_*f*_ increased: *Estimate* = 0.028, *SE* = 0.013, *z* = 2.086, *p* < 0.037 (see Fig. 3b). The total fixation duration on an image increased as the image’s *d*_*f*_ increased: *Estimate* = 0.091, *SE* = 0.007, *z* = 13.427, *p* < 0.001 (see Fig. 3c). There was also an effect of the paired image’s *d*_*f*_ on total fixation duration (*p* < 0.005) such that that monkeys spent less time fixating on images when they were paired with an image of greater *d*_*f*_. These findings demonstrate that macaques hold visual attention on small precise spatial areas more frequently on images with higher *d*_*f*_, on average for each fixed gaze hold a longer fixed gaze on images with higher *d*_*f*_, and overall spend more time in a fixed gaze on images with higher *d*_*f*_.

### Dwells

**Figure 4:**
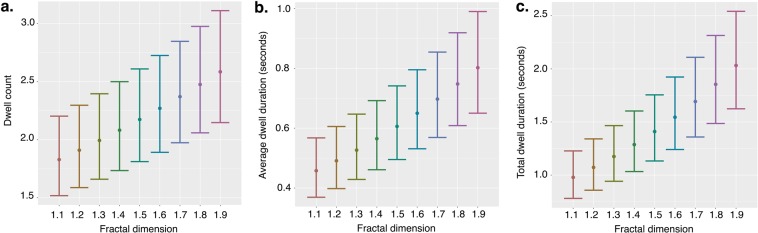
Dwell characteristics by fractal dimension. Macaques directed their vision (**a**) more frequently to images with greater *d*_*f*_, (**b**) on average scanned the images longer during each dwell, and (**c**) spent a longer total duration of time viewing images of greater *d*_*f*_. Estimated marginal means from the statistical model are depicted as points; error bars are the 95% confidence interval of these means. Paired *d*_*f*_ is held at 0.5 (See Supplementary Fig. S1 for complementary figures using raw data).

Macaques dwelled on images more often as the *d*_*f*_ increased, as indicated by an increase in dwell count across increasing *d*_*f*_: *Estimate* = 0.043, *SE* = 0.004, *z* = 9.789, *p* < 0.001 (see Fig. 4a). Animals evidenced longer dwells as the image’s *d*_*f*_ increased, such that the average dwell duration increased as *d*_*f*_ increased: *Estimate* = 0.070, *SE = *0.009, *z* = 8.166, *p* < 0.001 (see Fig. 4b). Similarly, the total duration of viewing an image, as indexed by total dwell duration on an *AOI* during a trial, also increased as *d*_*f*_ increased: *Estimate* = 0.091, *SE* = 0.007, *z* = 13.608, *p < *0.001 (see Fig. 4c). There were also an effect of the paired image’s *d*_*f*_ on dwell count (*p* < 0.001) and total dwell duration (*p* = 0.027), such that animals dwelled less frequently and for shorter durations on images when they were paired with images of greater *d*_*f*_. These findings demonstrate that macaques directed their visual attention to images with higher *d*_*f*_ more frequently, that on average their visual attention during a visit to an *AOI* was held for longer on images with higher *d*_*f*_, and overall they spent more time viewing images with higher *d*_*f*_ (see Fig. 1).

### Pupil diameter

**Figure 5:**
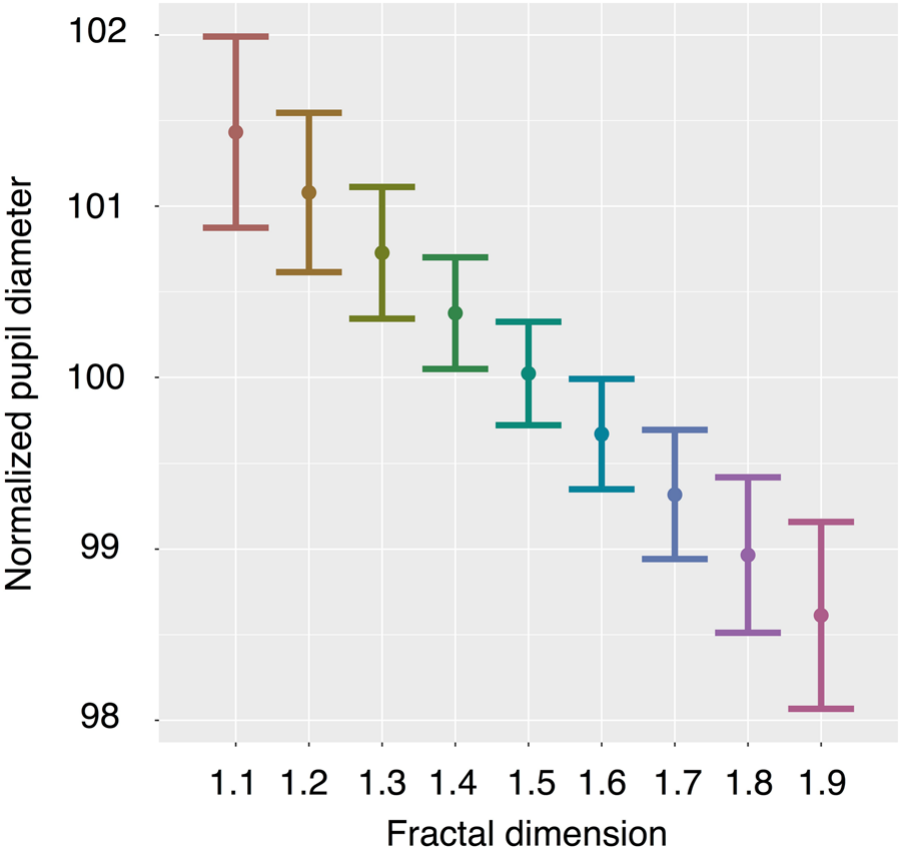
Pupil Diameter. Pupil diameter decreased with higher *d*_*f*_. To account for individual differences in pupil diameter we set subjects as a random effect in our statistical model. Here, for visualization purposes, we normalized pupil diameter based on the individual’s overall average across all stimuli and all trials. The average pupil diameter on a stimulus during a trial was divided by an individual’s average pupil diameter across all stimuli and all trials, then multiplied by 100. Thus, a 100 normalized pupil diameter would match the average across all trials, or 100% of average pupil diameter; greater than 100 is larger than average; less than 100 is smaller than average. Estimated marginal means from the statistical model are depicted as points; error bars are the 95% confidence interval of the mean. Paired *d*_*f*_ is held at 0.5 (See Supplementary Fig. S2 for a complementary figure using raw data).

Average pupil diameter decreased as *d*_*f*_ increased: *Estimate* = −0.24119, *SE* = 0.04271, *t value* = −5.648, *p* < 0.001 (see Fig. 5). We also compared this to a model that included the paired image dimension as a predictor (see Supplementary Materials for estimates of this model), but the model reported here is a better logical fit for the measure of pupil diameter as it is a physical measure of the eye while looking only at one *AOI*. (See also Supplementary Materials for estimates using non-normalized pupil diameter values.) These findings provide additional evidence that viewing behavior of macaques varies with the *d*_*f*_ of images.

### Attentional bias to higher fractal dimension

**Figure 6:**
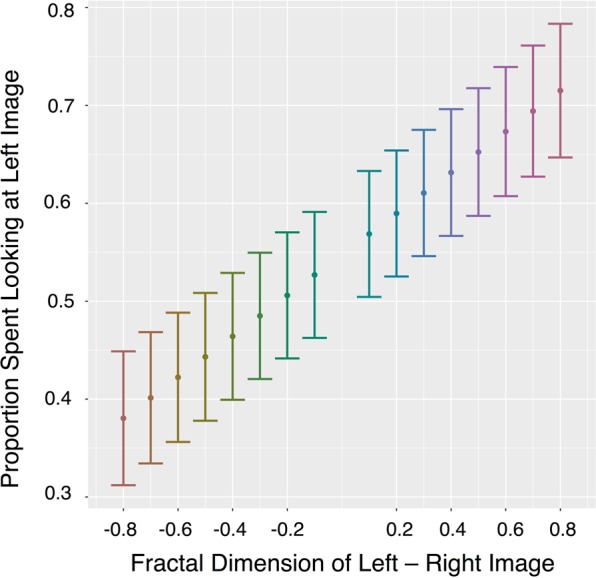
Attention bias. Bias scores depict attentional preference for images presented on the left side of the computer screen, computed as the total dwell duration on the stimulus presented on the left divided by the total dwell duration on both stimuli. This bias was calculated for each trial. Bias is plotted as a function of the difference in *d*_*f*_ between the left and right stimuli. Bias increased as the difference between *d*_*f*_ of the images increased. ±0.8 is the largest difference between the fractal dimensions of visual stimuli (i.e., 1.1 versus 1.9) and 0 is the smallest difference between the fractal dimensions of the visual stimuli (i.e., when stimuli are the same *d*_*f*_, not included in this analysis). Positive x-axis values indicate that the image presented on the left side of the screen had higher *d*_*f*_, while negative x-axis values indicated that the image presented on the right side of the screen had higher *d*_*f*_. Proportions greater than 0.5 on the Y axis indicate a bias towards the image presented on the left, while proportions smaller than 0.5 indicate a bias towards the image presented on the right. Estimated marginal means of statistical model are depicted as points; error bars are the 95% confidence intervals of these means (See Supplementary Fig. S3 for a complementary figure using raw data).

Humans report aesthetic preferences for curves and contours with certain fractal dimensions, though specific preferences appear to vary across studies that differ in stimuli types and experimental methodologies^22–25^. We investigated the possibility that monkeys might also have preferences for viewing visual information of particular *d*_*f*_ by computing an index of their attentional bias towards one stimulus relative to the difference between its *d*_*f*_ and the *d*_*f*_ of the stimulus with which it was paired. Macaques’ attentional bias toward higher *d*_*f*_ increased as the difference in *d*_*f*_ between images increased: *Estimate = *0.0209, *SE = *0.0022, *t*(1464) = 9.702, *p* < 0.001 (see Fig. 6). This suggests that beyond the ability to discriminate between *d*_*f*_ and overall greater attention directed towards images with higher *d*_*f*_, macaques may prefer viewing higher dimension fractal images – at least those up to *d*_*f*_ = 1.9 (we did not test uniform fields at *d*_*f*_ = 2.0, since there are no contours to which to attend).

## Discussion and Conclusion

This study demonstrates that rhesus monkeys, like humans, can detect and spontaneously discriminate between visual stimuli that differ in terms of *d*_*f*_. The different visual attention metrics that we analyzed in this experiment are thought to index different components of attention, and thus their consistent variation by *d*_*f*_ demonstrates the robust nature of this effect.

Generally, in eye-tracking data, fixations provide a metric or index of information processing, since acquisition of information about visual patterns occurs during fixations^54–56^ and sensitivity to visual input is suppressed during saccades (eye movements between fixations)^57,58^. Fixation density is therefore typically greater on regions of a scene that have more visual or semantic information present within them^59^. Since visual information is acquired during fixations, they are also generally thought to be related to (or be an index of) the allocation of attention to an image^54^, although, fixation on a region may not account for covert attentional processes to other regions^54,60^. More frequent and longer fixations to stimuli of higher *d*_*f*_ indicate overt visual attention was directed more towards stimuli of higher *d*_*f*_. This may be because images of higher fractal dimension contain more information that is distinguishable to macaques, and thus macaques spent more time visually processing these images.

Dwells are quantified at the unit of separate visits of one’s gaze to an area of interest and encompass any number and duration of fixations and saccades that occur on the AOI during that visit. While information acquisition is likely suppressed during saccades, certain types of cognitive processing may not be suppressed; specifically, dorsal-stream processes relating to spatial information are thought to be suppressed while ventral-stream processes related to object identification are not^56^. Thus, dwell durations index the total time scanning an image that includes both the duration of processing occurring while fixating on the AOI, and the duration between fixations on the AOI, that potentially have differential saccade durations, blink counts, and blink durations, that could include additional ventral-stream processing. Dwell counts index the number of times vision was directed at an AOI during a trial, which can vary independently from fixation count or duration, and indicate that macaques directed their vision to stimuli of higher *d*_*f*_ more often. More frequent and longer dwells on stimuli of higher *d*_*f*_ indicate macaques directed their vision more often to stimuli of higher *d*_*f*_ and scanned these images for longer, suggesting their attention was more readily captured and held by higher fractal dimensions (for 1 < *d*_*f*_ < 2) (see Fig. 1).

Measures of various fixation and dwell attributes (e.g., counts versus average duration) could hypothetically have subtly different interpretations regarding information processing and attention, though would require some speculation as they have been found to vary with a variety of stimulus characteristics and mental processes in humans^61^. However, the consistent increase in all of these variables with higher *d*_*f*_ in our data set points towards differential processing of images by *d*_*f*_, and more overt attention directed towards images of higher *d*_*f*_. That is, these measures of visual attention all confer the same take home message: monkeys are sensitive to variation in contour *d*_*f*._

We also demonstrated that monkeys’ dark-adapted pupil diameters decrease as fractal dimension increased. These results offer further support for the argument that how macaques perceive these images varies by the images’ *d*_*f*_ and, thus, that the macaque visual system is sensitive to the unique quality of visual patterns that is indexed by *d*_*f*_. Like other visual attention metrics, pupil diameter has been reported to vary with a variety of stimuli characteristics, mental processes, and affective states in humans^61–63^. The largest changes in pupil size occur in response to levels of illumination^62,64^, but since light levels were consistent during trials and stimuli had the same overall contrast and luminance in our study, the observed changes in pupil diameter were driven by other factors. Pupil diameter is modulated by parasympathetic nervous system activity (which constricts the pupil) and sympathetic nervous system activity (which dilates the pupil)^41,65^. While testing in darkness is thought to lessen parasympathetic contributions, processing tasks can influence pupil diameter through both pathways in light or dark adapted conditions^65^. Available evidence suggests that the two systems interact in complex ways across different affective and cognitive states^44,62,65^. Nevertheless, existing evidence consistently demonstrates that pupil diameter increases with increased mental effort or more mental workload^61,64,66–69^ and, in some cases, then decreases after a point of mental overload^70^. Pupil diameter has even been shown to increase with difficulty of nonvisual tasks^71^. Similarly, pupil diameter is frequently used as an index of affective states, namely stress, such that pupil diameter is larger with increased mental stress^72,73^. Thus, changes in pupil diameter may correspond to an ‘intensity’ aspect of attention^42,68,69,74^. Smaller pupil diameters while fixating on images of higher *d*_*f*_ indicate differential activation of the autonomic nervous system that is consistent with differential cognitive processing and support the possibility that viewing images of higher *d*_*f*_ may be less attentionally effortful.

Since macaques had larger dwell and fixation counts and durations when viewing informationally rich higher-dimension fractals, one might expect the opposite of the observed pupil diameter results – as more attention is directed towards images with higher *d*_*f*_, that pupil diameter would also increase, because processing more information with finer detail would require more intensive mental effort. Given the similarity of our stimuli and naturally occurring patterns macaques have likely encountered before (i.e., their ecological relevance), it is unlikely that these stimuli were so information rich that the negative relationship between *d*_*f*_ and pupil diameter is due to mental overload. Thus, while macaques spent more time visually processing higher dimension fractal images, it is unlikely they are more difficult for them to visually process.

Unlike humans who typically self-report preferences towards low-to-mid-range *d*_*f*_ in statistically self-similar stimuli of similar black and white fractal boundary contours^23^, monkeys in our experiment showed attentional biases towards higher dimension fractals across the entire range up to *d*_*f*_ = 1.9. The consistent bias of attention towards images with relatively higher *d*_*f*_ across the whole range of images suggests that monkeys’ attention was consistently captured by the image that contained the highest *d*_*f*_, and that this bias was greater when the difference in *d*_*f*_ between images increased. Efficient processing of and attentional biases for particular patterns may relate to the statistical regularities of an animal’s natural environment since sensory systems evolved and developed to perceive information readily in these environments^3,75^. One previously proposed hypothesis relating human preferences for mid-range *d*_*f*_ is that this range is commonly seen in nature^23,76^ and there may be a “resonance” while viewing – that is, a matching between the pattern of the visual stimulus and the pattern of human eye movements^23^. This is consistent with the premise that sensory system function will be related to statistics of the natural environment since they evolved to efficiently capture information in their environment^2–7^. While such environmental or physiological factors might differ across species, to conclude that monkey and human preferences of fractal dimensions truly differ, we will need to conduct experiments with similar design and data in both macaques and humans. These tasks give a clear future direction for this research program. Future work employing additional physiological measures and both fractal and non-fractal stimuli can address whether or not fractal structure actually allows for less-effortful processing of informationally rich stimuli, and if so, by means of species-typical eye trajectories^23^ or efficient cognitive processing of this type of fractal pattern^77,78^.

Sensitivity to fractal dimension could have important implications for a monkey’s interaction with his or her habitat, since fractals are so prevalent in nature. This idea is consistent with current standards for zoo animal habitats in which zoos have shifted towards creation of more naturalistic environments for captive animals^79–83^. Such naturalistic approaches strive for a visually accurate representation of a species’ natural habitat^84^, guided by the idea that natural environments facilitate normal behavior patterns^79,83^. For instance, when moved to a naturalistic island from laboratory cages, chimpanzees exhibited lower rates of stereotyped and self-directed behavior^83^, behavioral indices of stress. Similarly, mandrills moved from an indoor tiled enclosure to a naturalistic outdoor vegetated enclosure had activity budgets that more closely paralleled wild Mandrills^85^. However, given the expense of building new exhibits, it is important to understand more about animal-environmental interactions and understand which environmental features are most important for enhancing species typical behaviors and welfare^82^. Generally, little is known about which specific characteristics of a “natural” environment contribute to the welfare of a given species and, therefore, are most important to emulate. Considering what types of information are perceivable and preferred by a species is vital to successfully implementing a naturalistic approach to habitat design.

For humans, it is also the case that naturalistic environments may be beneficial. For example, being outdoors^86,87^ and viewing natural scenes^88,89^ are thought to reduce or help one recover from stress. A link between fractal dimension of stimuli and well-being has been suggested as a mechanism for this – viewing images of low-to-mid-range *d*_*f*_ curves induced the highest frontal alpha brain activity in humans, which has been associated with wakeful relaxation^90,91^. Since we determined that *d*_*f*_ is an index of pattern self-similarity to which monkeys are sensitive, stress responses to viewing such stimuli should be further investigated. Considering the visual patterns in environments may therefore be an important design choice for improving captive animal welfare or designing more stimulating environments for laboratory animals when a fully natural habitat is not possible. While our results provide clear evidence that stimuli that vary in fractal dimension are differentially attended to, future studies will need to employ choice tests to confirm that macaques have preferences for particular patterns in their habitat. Further, inclusion of additional physiological measures will allow us to determine if monkeys’ preferred *d*_*f*_ produces physiological responses indicative of welfare enhancement similar to those proposed in humans.

Additionally, *d*_*f*_ of boundary contour is just one modality of visual complexity – to paint a full picture of how animals process complex information it will be important to expand investigations to include different statistical modalities (e.g., texture^33,92^) and other complexity indices (e.g., types of entropy^12^ and structural complexity^12^ expressed visually). Also, some variability in human performance of *d*_*f*_ discrimination has been linked to cognitive abilities to encode information^19^. Since analyses of fixation count, average fixation duration, dwell count, average dwell duration, pupil diameter, and attention bias all revealed the random effect of the individual to be significant (all *p* < 0.001), and because pupil diameter was not correlated to *d*_*f*_ in the same direction as other attentional measures, this experimental framework can be used be explore between-animal individual differences and cross-species differences in information processing abilities. Finally, to determine that sensitivity to *d*_*f*_ is indeed evolutionarily conserved in primate or mammalian vision, future studies will need to assess the degree of proximate developmental effects by investigating animal sensitivity to *d*_*f*_ at different developmental stages as related to their visual environment during development, and also investigate a wider range of species.

Our demonstration that macaques differentially process and have biased attention towards images with greater *d*_*f*_ extends research on the perception of complex visual patterns to a larger evolutionary framework by showing that human sensitivity to *d*_*f*_ is shared with other primate species. This experiment, and subsequent future studies, may further our quantitative understanding of the sensory self-worlds of other species by isolating specific properties of the environment animals are capable of sensing or predisposed to notice. Understanding basic mechanisms of visual pattern information processing is critical for comparing how attentional systems are similar and dissimilar from humans, and for identifying how vision systems evolved. Perhaps equally important is the idea that basic research on complex information processing may ultimately lead to designing stimulating captive environments for animals that allow them to process and navigate through stimuli that are well-tuned to their own sensory systems. Thus, *d*_*f*_ and companion measures, such as structural complexity, that describe how information is organized in natural patterns may be key to the evolution of pattern recognition by sensory systems and, more broadly, to how nonhumans experience the world.

## Supplementary information

Supplemenatry Information

## Data Availability

The dataset will be made available on osf.io upon acceptance of the manuscript.
