# Supplementary material for: Macaques preferentially attend to visual patterns with higher fractal dimension contours
Source: Sci Rep. 2019 Jul 22;9:10592. doi: 10.1038/s41598-019-46799-0 (PMC6646383; doi:10.1038/s41598-019-46799-0)
Supplement: Supplementary file 1 — Supplemenatry Information [file 41598_2019_46799_MOESM1_ESM.docx]

***Supplementary Material for:***

**Macaques preferentially attend to visual patterns with higher fractal dimension contours**

Kelly R. Finn, James P. Crutchfield, & Eliza Bliss-Moreau

**Supplementary Results**

*Additional Pupil Diameter Models*

Best-fit models for pupil diameter differed in AIC by <1, and we chose the model that did not include paired image dimension because is a better logical fit for the measure of pupil diameter as it is a physical measure of the eye while looking only at one AOI. Results for the model including paired image dimension showed similar results. Average pupil diameter decreased as *d_f_* increased. Image :*_._ Estimate* = -0.34978, *SE* = 0.05893, *t value* = -5.936, *p* < 0.001; Paired image: *Estimate* = -0.14553, *SE* = 0.05911, *t value* = -2.462, *p* = 0.0139. We used within-subject normalized average pupil diameter as it accounts for individual differences in pupil physiology, and is an established normalization technique in the literature ^1,2^. We also ran models of non-normalized average pupil diameters, which also controls for individual differences in baseline pupil diameter using Subject as a random effect, but not individual differences in pupil reactivity or range of dilation. The best-fit model for non-normalized pupil diameter did not include paired image dimension and yielded similar results. Average pupil diameter decreased as *d_f_* increased:*_._ Estimate* = -0.24119, *SE* = 0.04271, *t value* = -5.648, *p* < 0.001.

**References**

1. Machado, C. J., Bliss-Moreau, E., Platt, M. L. & Amaral, D. G. Social and nonsocial content differentially modulates visual attention and autonomic arousal in rhesus macaques. *PLoS One* **6,** e26598 (2011).

2. Geng, J. J., Blumenfeld, Z., Tyson, T. L. & Minzenberg, M. J. Pupil diameter reflects uncertainty in attentional selection during visual search. *Front. Hum. Neurosci.* **9,** 435 (2015).

**Supplementary Figures**

*Additional Figures Using Raw Data*

**Supplementary Figure S1: Fixation and dwell characteristics by fractal dimension.** (a) Macaques fixated on images with greater *d_f_* more times during a trial, (b) on average fixated longer during each fixation, and (c) spent a larger total duration of time fixating on images of greater *d_f_*. Means are depicted as points; error bars are the standard error of the mean. Macaques directed their vision (d) more frequently to images with greater *d_f_*, (e) on average scanned the images longer during each dwell, and (f) spent a longer total duration of time viewing images of greater *d_f_*. Means from the raw data are depicted as points; error bars are the standard error of these means.


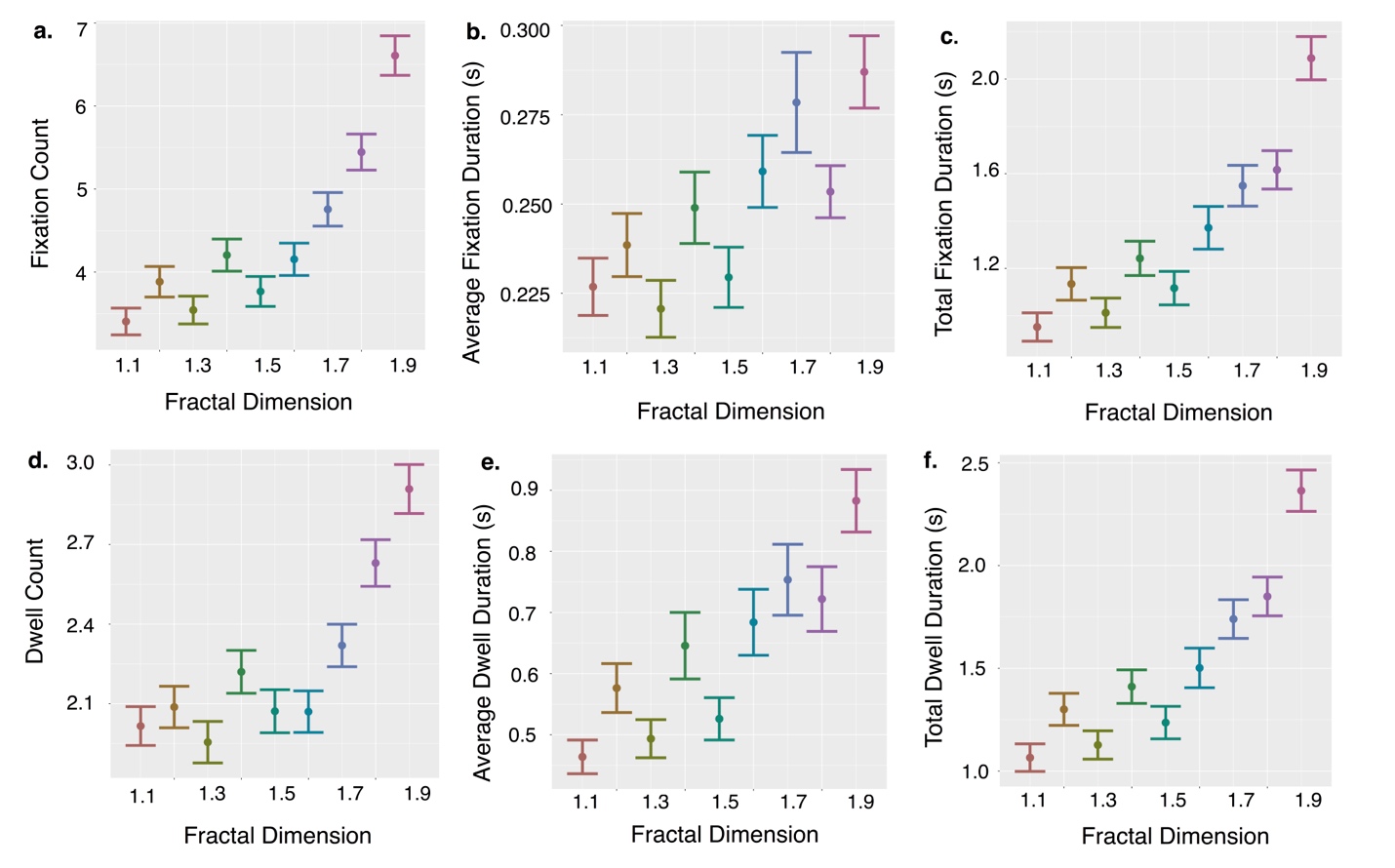


**Supplementary Figure S2: Pupil Diameter.** Pupil diameter decreased with higher *d_f_*. To account for individual differences in pupil diameter we used a within-subject normalization of average pupil diameters. The average pupil diameter on a stimulus during a trial was divided by an individual’s average pupil diameter across all stimuli and all trials, then multiplied by 100. Thus, a 100 normalized pupil diameter would match the average across all trials, or 100% of average pupil diameter; greater than 100 is larger than average; less than 100 is smaller than average. Means from the normalized raw data are depicted as points; error bars are the standard error of these means.

**
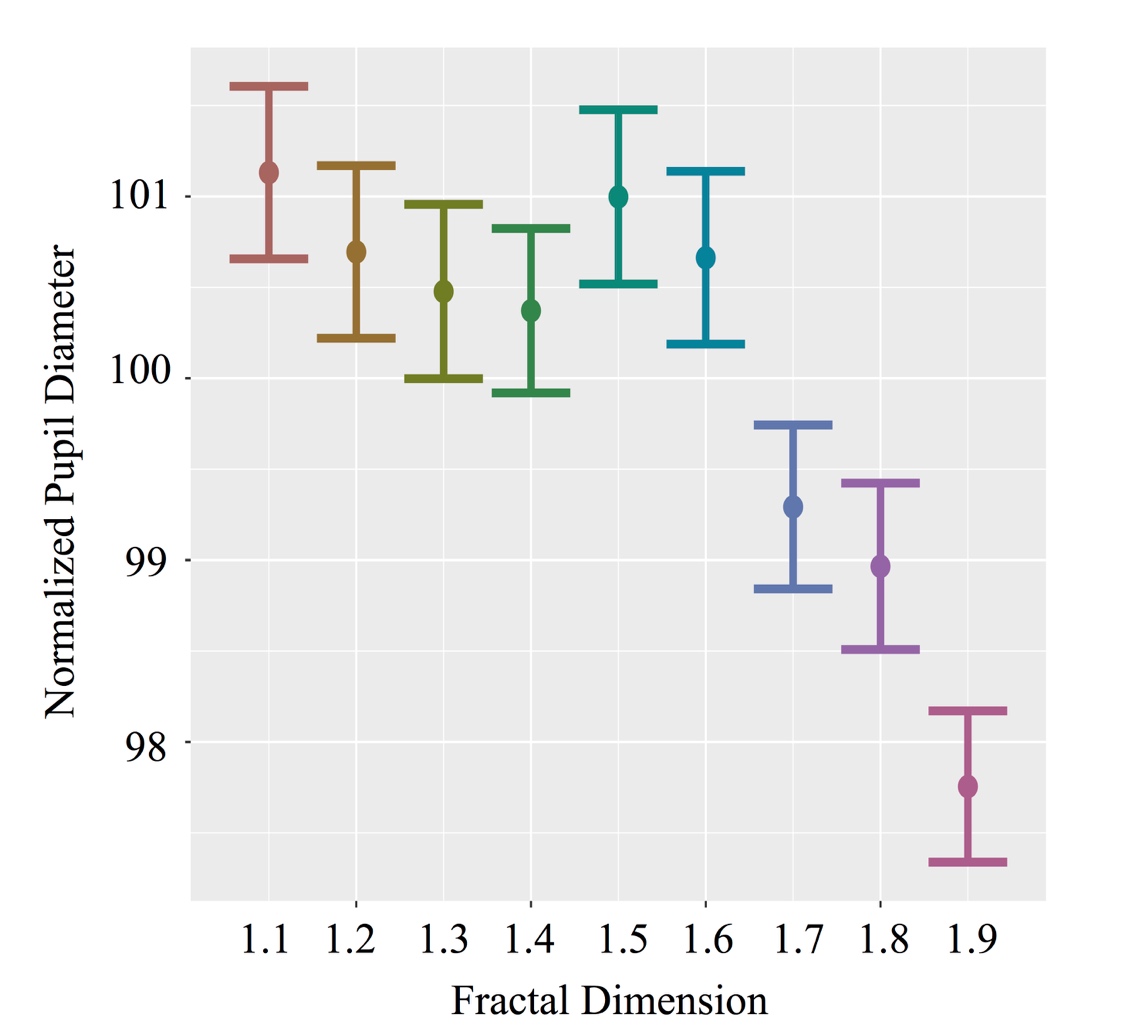
**

**Supplementary Figure S3: Attention bias.** Bias scores depict attentional preference for images presented on the left side of the computer screen, computed as the total dwell duration on the stimulus presented on the left divided by the total dwell duration on both stimuli. This bias was calculated for each trial. Bias is plotted as a function of the difference in *d_f_* between the left and right stimuli. Bias increased as the difference between *d_f_* of the images increased. ±0.8 is the largest difference between the fractal dimensions of visual stimuli (i.e., 1.1 versus 1.9) and 0 is the smallest difference between the fractal dimensions of the visual stimuli (i.e., when stimuli are the same *d_f_*, not included in this analysis). Positive x-axis values indicate that the image presented on the left side of the screen had higher *d_f_*, while negative x-axis values indicated that the image presented on the right side of the screen had higher *d_f_*. Proportions greater than 0.5 on the Y axis indicate a bias towards the image presented on the left, while proportions smaller than 0.5 indicate a bias towards the image presented on the right. Means of the raw bias scores are depicted as points; error bars are the standard error of these means.

**
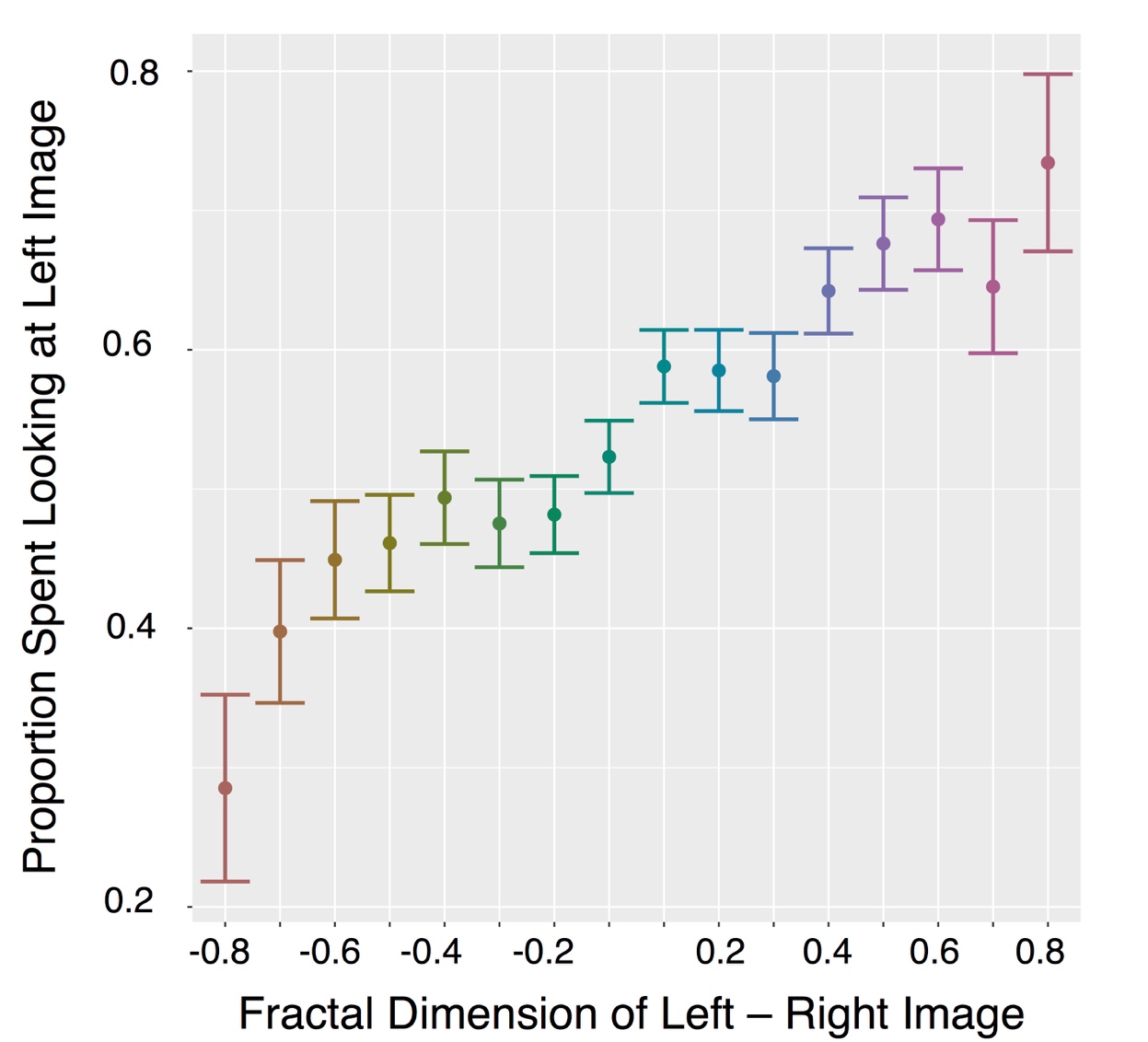
**
